# Professionals’ Attitudes After a Seclusion Reduction Program: Anything Changed?

**DOI:** 10.1007/s11126-012-9222-6

**Published:** 2012-05-19

**Authors:** P. S. Mann-Poll, A. Smit, M. van Doeselaar, G. J. M. Hutschemaekers

**Affiliations:** 1 Pro Persona Mental Health Care, Pro Persona Centre for Education and Science (ProCES), Tarweweg 6, Nijmegen, The Netherlands; 2 Pro Persona and Academic Centre of Social Sciences, Radboud University, Nijmegen, The Netherlands

**Keywords:** Professionals, Attitudes, Seclusion, Change, Mental health care

## Abstract

Changing professionals’ attitudes toward seclusion is seen as an important condition to reduce its use. The purpose of this study was to determine whether professionals from a mental health institute in the Netherlands changed in their attitudes toward seclusion after implementation of a multifaceted seclusion reduction program. Professionals working on four acute admission wards filled in the Professional Attitudes Toward Seclusion Questionnaire (PATS-Q) before and after a seclusion reduction program. Changes were analyzed by comparing mean scores on the PATS-Q. After the program, professionals scored significantly higher on ‘ethics’ and ‘more care’. As expected, no change occurred on ‘reasons’ for the use of seclusion. In addition, no significant changes were found on ‘confidence’, ‘better care’ and ‘other care’. Significant changes in professional attitudes concerning the ethics of using seclusion and involving issues of more care were observed after a seclusion reduction program. Mental health professionals moved in the direction of ‘transformers’, indicating an increased criticism of the practice of seclusion and increased willingness to change their own use of seclusion.

## Introduction

The use of seclusion is increasingly seen as controversial and reducing its use in mental health care is a priority health policy issue in many Western countries including the Netherlands [1, 2]. Seclusion in this study is defined as locking a patient into a special, unfurnished room which he or she cannot leave without permission by staff [3, 4]. Despite the controversy, seclusion continues to be a commonly used intervention in Dutch psychiatric wards, while no scientific evidence is available for the therapeutic effects of its use [5, 6]. Although it is difficult to find reliable data of seclusion rates in the Netherlands, on average, still one in four hospitalized patients will experience a seclusion episode [7]. Furthermore, a study evaluating different European cultures and perspectives toward the use of seclusion showed that Dutch professionals, compared to those in Finland and the UK, are less in favour of using seclusion than their colleagues in Finland, but more than their colleagues in the UK [8].

According to Quinn (1996) a ‘deep change’ is necessary for a sustainable change in actual practice [9]. Deep change defined as: “A change that goes beyond surface structures or procedures to alter professionals’ beliefs, norms and social interactions” is adapted from educational research [10]. Huckshorn (2006) [11] translated deep change to psychiatric practice and the aim to consequently reduce the use of seclusion and saw a change in attitude of professionals toward seclusion as an important condition to achieve this goal. Professional attitudes and ward culture are often mentioned as important determinants in the reduction of the use of seclusion in mental health care [12–19]. Several educational programs to help staff learn about different ways to handle violent or disturbed patients seemed to be successful in decreasing seclusion rates [13, 17, 19–22]. Implicitly, the success was related to an attitude change of professionals although these studies did not actually assess these attitudes, let alone the attitudinal change. In general, empirical evidence concerning the way to achieve attitude change of mental health professionals toward the use of seclusion is lacking [23–25]. Although the attitude change toward seclusion as a separate subject has never been measured, two studies in which changes in professionals’ attitudes toward coercive measures in general were found. Bowers et al. (2004) assessed the change of attitudes of nurse students during their training repeatedly over time using the ACMQ (Attitude Toward coercive Measurements Questionnaire) [26]. Pellfolk et al. [27] used the PRUQ (Perceptions of Restraints Use Questionnaire) in geriatric care. Contrary to expectations, both studies did not find any change in attitudes after the training.

In the Netherlands, van Doeselaar et al. [28] developed and used the Professionals’ Attitude Toward Seclusion Questionnaire (PATS-Q), which is also used in this study before and after a seclusion reduction program [29, 30]. This questionnaire is described in more detail in the methods section.

van Doeselaar et al. [28] conducted a cluster analysis, using PATS-Q scores of 540 Dutch professionals and identified three types of professionals; maintainers, doubters and transformers. The group maintainers scored low on average for all attitude sub scales. The doubters, contrary to the other types, saw seclusion primarily as a therapeutic intervention rather than that they questioned its use. The third group, the transformers, showed relatively little confidence in seclusion. For transformers, ethical considerations dominated the picture. Also, they were strongly in favour of finding alternatives for seclusion and showed a greater willingness to change existing practices, even though their reasons for using seclusion did not differ that much from the doubters and the maintainers.

Building on the results of van Doeselaar [28], we expected that after participating in a seclusion reduction program (SRP) from 2004 till 2008, professionals would show a significant change in their attitudes on the PATS-Q. Moreover, we hypothesized that professionals would change their attitude in the direction of the ‘transformers’, because of the three different types determined, especially these professionals wanted to change the current seclusion practice. Thus, the desired attitude change would be shown as the following PATS-Q outcome:

- A higher score on the ethics and a lower score on the confidence sub scale.
- No changes on any of the three sub scales for reasons, because the group of ‘transformers’ could not be distinguished from the other groups (of doubters and maintainers) on these scales.
- A higher score on all three subscales for alternatives, especially for other care in which the transformers distinguished themselves.

In summary: after implementation of the SRP, we expected to find a change on five of the eight sub scales on the PATS-Q in the desired direction of the ‘transformers’.

The aim of this study was to determine changes in professional attitudes after a seclusion reduction program (SRP) by answering the following research questions:

1. Did professionals change their attitudes after the SRP?
2. Given the goal of the SRP: did professionals change their attitudes in the desired direction of the ‘transformers’?

## Methods

Professionals’ attitudes toward seclusion were assessed on four acute admission wards (three wards for adults 23–60 years and one for the elderly >60 years) both at the start of the SRP in 2004 and at the end in 2008. One of these wards started at the end of 2002 with the development and implementation of a SRP program [29]. Successively, from 2003 onwards, the other three admission wards followed and by 2008, the program was implemented in the whole institute [30]. Teams of each ward were free to choose their own package from the interventions offered in the program as described below.

## The Seclusion Reduction Program (SRP)

The main elements of the SRP used in this institute were:

1. Vision development of multidisciplinary teams (including the psychiatrist, mental health nurses, the psychologist and the activity therapist),
2. Training (on the job) (risk taxation, pro-active working, approaching patients and making contact especially when this is hardly possible),
3. Weekly clinical supervision meetings by an external supervisor,
4. Monitoring and feedback of seclusion rates,
5. Exchange with other (national and international) acute admission wards and visiting national and international work conferences directed on the subject.

## Instruments

To investigate the attitude changes of professionals toward seclusion, we used at two time points the validated Dutch Professionals Attitudes Toward Seclusion Questionnaire (PATS-Q) before and after the SRP [28]. The PATS-Q is sensitive to change and therefore appropriate to provide insight in the change of professional attitudes.

**Fig. 1:**
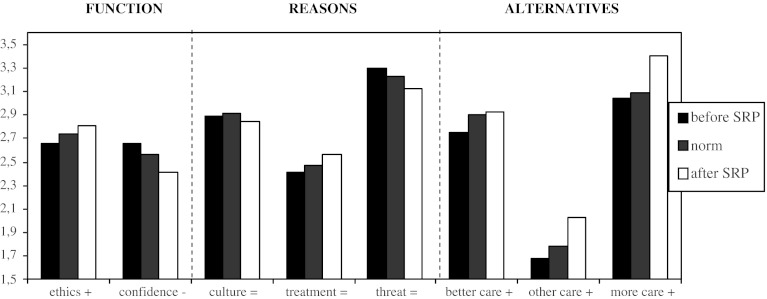
Graphic change in professionals’ attitudes toward seclusion

Aside from a few socio-demographic questions, the questionnaire consists of three main scales, totalling eight subscales (see Fig. 1). The first main scale: ‘Function’ (with two subscales: ethics and confidence) consists of 14 statements on the different functions of seclusion, such as: ‘a form of treatment’, ‘a necessary evil’ and as having an unjustifiable impact on patients. The second main scale: ‘Reasons’ (with three scales: culture, treatment and threat) is built from 17 statements on possible causes of seclusion, including violence and the prevailing ward culture. The third main scale: ‘Alternatives’ (with three sub scales: better care, other care, and more care) contains a list of 12 alternatives for seclusion such as ‘improving protocols’, ‘make ward rules more flexible’, and ‘more nurses’. Respondents were asked to rate statements on a four point Likert scale, ranging from (1) strongly disagree to (4) strongly agree.

The internal consistency (Crohnbach’s alpha) of the subscales ranged from good 0.84 (better care) to satisfying 0.68 (ethics). Additional data on psychometric properties of the PATSQ have been presented in a previous paper [28].

## Data Analysis

We controlled our population, by comparing attitudes of professionals of this specific institute to those of Dutch professionals in general using an independent *T* test. At the start of the SRP in 2004, no significant differences in attitudes towards seclusion between our research population and the norm scores of Dutch mental health professionals in general were found on any of the PATS-Q scales (*p* > 0.05; data not shown). Norm scores were developed by using the mean scores of a large sample of Dutch professionals collected in various working environments and presented in a previous study [28].

Successively, changes were analyzed by comparing mean ward PATS-Q scores of 2004 with those of 2008 using a paired sample *T* test (see Table 1). To investigate if the change occurred in the desired direction, we designed a graphic bar chart (see Fig. 1). Finally, to investigate if the attitude change was significant, we computed deviation scores (distances from the norm scores) for 2004 and 2008 and compared them using a paired sample *T* test (see Table 2). All calculations were performed using SPSS 16.0.

**Table 1:** Assessment of professionals’ attitude before and after the SRP

PATS-Q sub scales	Ethics mean (SD)	Confidence mean (SD)	Culture mean (SD)	Treatment mean (SD)	Threat mean (SD)	Better care mean (SD)	Other care mean (SD)	More care mean (SD)
Scores 2004	**2.66 (0.13)**	2.66 (0.22)	2.89 (0.16)	2.41 (0.28)	3.30 (0.19)	2.75 (0.28)	1.68 (0.27)	**3.04 (0.12)**
Scores 2008	2.81* (0.18)	2.41 (0.33)	2.84 (0.32)	2.56 (0.28)	3.12 (0.51)	2.93 (0.25)	2.03 (0.43)	3.40* (0.19)

Bold values indicate a significant change on these subscales

* *p* < 0.05

**Table 2:** Mean and SD deviation scores

PATS-Q sub scales	Ethics mean (SD)	Confidence mean (SD)	Culture mean (SD)	Treatment mean (SD)	Threat mean (SD)	Better care mean (SD)	Other care mean (SD)	More care mean (SD)
Deviation scores 2004	**−0.2 (0.34)**	0.35 (0.42)	**−**0.04 (0.30)	**−**0.13 (0.59)	0.20 (1.13)	**−**0.29 (0.54)	**−**0.15 (0.36)	**−0.11 (0.26)**
Norm score	**2.74 (0.38)**	2.56 (0.42)	2.91 (0.53)	2.47 (0.48)	3.23 (0.45)	2.90 (0.52)	1.79 (0.73)	**3.09 (0.46)**
Deviation scores 2008	0.17* (0.48)	**−**0.35 (0.78)	**−**0.14 (0.61)	0.18 (0.61)	**−**0.25 (1.13)	0.05 (0.49)	0.33 (0.59)	0.67* (0.21)

Bold values indicate a significant change on these subscales

* *p* < 0.05

## Results

### Sample

Response rates were 79.5 % (35 of 44) in 2004 and 60 % (24 of 40) in 2008. There was a significant difference in gender (*p* = 0.001) of the respondents: At the first measurement, 60 % were female, while this percentage was 41 % at the second measurement. Also, the respondents in 2008 had significantly more experience with the use of seclusion: 5–10 years versus 2–5 years in 2004 (*p* = 0.024). Most respondents were between 40 and 50 years old (2004 = 41 %; 2008 = 32 % *p* = 0.164). Respondents were predominantly nurses, (2004 = 88.6 % and 2008 = 95.5 %) a small minority (2004 = 11.4 % and 2008 = 4.5 %) were medical doctors and/or psychiatrists. Most respondents in 2004 (2004 = 47.1 %) were personally involved with seclusion one to four times a month (47.1 %) and in 2008 less than once a month (50 %). There were no statistically significant differences between the variables age, discipline and personal involvement in the use of seclusion (*p* > 0.05).

### Change

Two of the eight sub scales showed statistically significant differences; ‘ethics’, part of the function scale (mean difference 0.14; *p* = 0.04) and ‘more care’, part of the scale that involved alternatives for seclusion (mean difference 0.36, *p* = 0.01) (see Table 1). Scores on ethics and confidence in seclusion were more strongly opposed to each other in 2008, which is a typical phenomena for the group of ‘transformers’. In line with our hypotheses, we did not find any significant changes in the reasons for seclusion scales. In the opinion of the professionals, threat remained the most dominant reason for the use of seclusion, followed by ward culture.

The higher scores on ethics in 2008 indicate that professionals became more reflective and ask themselves more ethical questions about the necessity and the desirability of the use of seclusion. However, contrary to our hypothesis, no significant changes were found for confidence and alternatives like other care, and better care. Professionals did not change in their search for alternatives like ‘making ward rules more flexible’ or ‘closing seclusion rooms’. Apparently, professionals were even more convinced of the benefits of utilizing ‘more care’ alternatives, like more nurses, more medication and earlier risk taxation, than of ‘out of the box’ thinking alternatives after the SRP.

### Direction of Change

Figure 1 shows the graphic change in direction of professionals’ attitudes compared to the general Dutch norm scores (the symbols behind the scales show the desired direction of change). As shown, the attitude changes occurred in the desired direction of the ‘transformers’.

In order to determine whether the change was significant, we computed deviation scores (the relative distance of the scores from the norm before and after the implementation of the SRP). Comparing these scores, significant changes on the sub scales ‘ethics’ (*p* = 0.04) and ‘more care’ (*p* = 0.007) in the direction of the transformers were observed. Thus it seems that mental health professionals in these teams not only changed their own opinion about seclusion after the SRP, but their attitudes also changed in comparison to their Dutch colleagues. In other words: after the SRP, professionals of this institute had a more critical attitude towards the use of seclusion than their Dutch colleagues in general. Furthermore ‘more care’ (i.e. more nurses, more medication and earlier risk taxation) became the main alternative for the use of seclusion. Thus, although the graphic change of ethics, confidence, better care, other care and more care seemed to change in the desired direction of the group ‘transformers’ (see Fig. 1), the change of ethics and more care reached the level of significances (see Table 2).

## Discussion

With PATS-Q assessments before and after the SRP, this is the first study that provides empirical evidence regarding professionals’ changes in attitudes toward the use of seclusion in a mental health care setting over time. Significant changes were observed on two sub scales; within the main scale Function (consisting of sub scales ‘ethics’ and ‘confidence’), the sub scale ‘ethics’ showed a significant change in the desired direction. Likewise, within the main scale Alternatives (containing sub scales ‘better care’, ‘more care’ and ‘other care’), the sub scale ‘more care’ showed a significant improvement. As expected, no changes were observed on any of the three subscales concerning reasons for using seclusion. Overall, findings showed a shift in professionals’ attitudes in the direction of the ‘transformers’.

A recent review by Happell and Harrow [31] concluded that the majority of mental health nurses still consider the use of seclusion to be an important strategy for the management of violence and aggression, even after several initiatives to reduce seclusion [32]. Given this result, could we actually expect an attitude change after the implementation of the SRP? In our view, this expectation was justified because the SRP was a multifaceted program principally focused on altering professionals’ beliefs and norms. The program consisted of vision development, training and supervision combined with regular feedback on actual use of seclusion, all of which are considered essential elements of successful elimination programs [13, 17, 19–22]. Moreover, we were able to specify the desired direction of attitude change by building on the results of the study by van Doeselaar et al. (2008) in which a correlation between willingness to change and type of professional was found.

We found that professionals changed their attitudes toward seclusion into more critical ones, despite their significant increased work experience. This is a remarkable finding, given that several studies investigating the impact of clinical work experience have shown the opposite; the more clinical work experience the more positive about the use of coercive measures [28, 33–35]. Nevertheless, scores on confidence in seclusion showed no significant decrease, and no shifts were observed regarding the use of alternatives described in ‘other care’ and ‘better care’. It seems that although professionals proved to be able to distance themselves somewhat from daily practice, they may feel unable to think in solutions other than those already existing.

van Doeselaar gave two possible explanations for becoming the type of ‘transformer’. The first one was of self-selection: ‘especially teams that were dissatisfied with the current situation and wanted to change, took part of a SRP and were willing to change’. The second explanation, which van Doeselaar could not definitively prove by her results, was that of becoming a ‘transformer’ as a consequence of participation in a SRP. In that way, professionals have become more critical of seclusion and have learned to distance themselves from prevailing professional practice. Given that professionals’ attitudes at this specific institute by start of the SRP did not differ from the attitudes of their Dutch colleagues in general, indicates that participation in the SRP itself resulted in a grown critical attitude toward the use of seclusion. Thus implementing a SRP leads to a change in professional attitudes. This is a hopeful result for policy makers and professionals who actively want to implement an attitude change toward the use of seclusion practice in mental health care.

Taking into account that professionals became more critical after the SRP, an important question still remains: is this attitude change of professionals a necessary condition for realizing a deep change in seclusion practice [12–18] or does a reduction in the use of seclusion automatically imply a change in attitudes [13, 17, 19–22]?

Also: an important question arises whether a complete attitude shift of all participating professionals into the desired direction of the ‘transformers’ will be necessary to realize a deep and sustainable change. It might be possible that we only need some ‘transformers’ working on an admission ward to lead the reduction in the use of seclusion. This is suggested by several policy studies [10, 16, 24].

To answer these questions, more differential methods and strategies to investigate change of professionals in mental health care practice are needed.

## Limitations

Due to the regulatory rotation of professionals in an acute admission setting, the individual respondents representing the wards participating in this study, changed over time. While this enables us to draw conclusions on a group level, it does not allow us to analyse changes on an individual level.

We cannot be certain if the individual respondents working on participating wards were also actively participating in the SRP.

Significant differences in gender and work experience with seclusion between the respondents in 2004 and 2008 were observed. However, van Doeselaar et al. (2008) investigated associations between type of professional and personal characteristics and found no correlations. Thus, we assume that these differences have no consequences for our main findings.

We only assessed the teams two times, before and after the SRP. In order to get more insight in the process of attitude change, further assessments over time are preferable.

## Conclusion

Significant changes in professional attitudes concerning the ethics of using seclusion, and involving issues of more care were observed after a seclusion reduction program. Mental health professionals moved partly in the direction of “transformers”, indicating an increased willingness to question and change their own seclusion practice.
